# Global Antimicrobial Stewardship: A Closer Look at the Formidable Implementation Challenges

**DOI:** 10.3389/fmicb.2016.01860

**Published:** 2016-11-16

**Authors:** John J. L. Tiong, Jason S. E. Loo, Chun-Wai Mai

**Affiliations:** ^1^School of Pharmacy, Taylor’s UniversitySubang Jaya, Malaysia; ^2^School of Pharmacy, International Medical UniversityBukit Jalil, Malaysia

**Keywords:** antimicrobial stewardship, antibiotic resistance, implementation challenges, global framework, action plans, health crisis

## Abstract

Antimicrobial stewardship (AMS) has been touted as one of the key strategies required in tackling worldwide escalation of antibiotic resistance. Although AMS has optimized antibiotic usage and reduced the incidence of resistance development in some regions, its full global potential has been curtailed by various AMS-impeding factors. This article seeks to highlight in a detailed perspective, the key challenges that hamper global AMS endeavors, some of which include the paucity of effective implementation strategies that cater for the challenging settings of developing nations, the slow response of governments, uncoordinated AMS activities as well as implementation fragmentation across different sectors and countries. The authors of this article call upon all stakeholders to pay attention to these seemingly obvious but often under-addressed problems. If left unresolved, this may render all current and future AMS initiatives pointless.

## Introduction

The worldwide escalation in antibiotic resistance has sparked fear of a looming post-antibiotic era (WHO, 2014). Such a global health crisis would render even common infections untreatable with the current cocktail of antibiotics available. With the recent discovery by Chinese scientists of a strain of *Escherichia coli* in local livestock resistant to the last resort antibiotic colistin, the prediction of an antibiotic apocalypse is somewhat reaffirmed (Liu et al., 2016). Of greater concern is the speed of trans border spread of such resistance which was reported in Europe only several months ago and has since reached the US (Spencer, 2015; Christensen, 2016). The worrying spread of drug-resistant infections is causing 700,000 deaths annually at present and if left unaddressed, it is projected to cause 10 million lives per year by 2050 with a cumulative economic cost of 100 trillion USD (O’Neill, 2016). The advocacy of rational use of antibiotics began forthwith the recognition of their inappropriate use being the key driver of resistance development (Goldmann and Huskins, 1997; WHO, 2002; Hashemi et al., 2013). As such, Antimicrobial stewardship (AMS) has emerged as one of the key strategies employed to minimize the development of resistance in order to preserve antibiotic efficacy in the light of dwindling antibiotic candidates in the pipeline. AMS-guided interventions have optimized antibiotic usage, reduced the incidence of resistance development and improved clinical outcomes in hospital inpatient settings as reported by the Cochrane Reviews (Davey et al., 2013). Nevertheless, it remains challenging to gauge the achievements of AMS from an international perspective since the global surveillance data pre- and post–AMS programs implementation are limited and the level of surveillance undertaken varies across different regions (WHO, 2015). Unfortunately, it is evident at present that the worldwide rate of antibiotic resistance development has shown no sign of slowing down whilst the global consumption of antibiotics continues to rise over the years (Boeckel et al., 2014; WHO, 2014). So what exactly did we miss? A closer look at the key problems plaguing current AMS initiatives can spur more ideas to further refine feasibility of AMS alongside the novel solutions proposed by many thus far, particularly in terms of improving the implementation aspects on a global scale. This article seeks to illustrate in a detailed perspective, the main implementation-impeding challenges which have curtailed the full potential of AMS. The authors of this article are aware that there may not be straightforward solutions to these issues although we strongly believe that improvements are attainable through progressive changes if these aspects are given due consideration in all proposed AMS plans in the future.

## Resources Limited Environments of Developing Nations

The AMS programs are a heterogeneous mix of system- and organizational-based interventions espoused internationally to address the antibiotic resistance epidemic (Doron and Davidson, 2011; NHS, 2015). Despite the numerous strategies set forth in AMS programs to contain the spread of resistance, there is unlikely a single ‘perfect’ approach in ensuring their implementation across the board, more so within the resource-poor environment of low- and middle- income countries (LMICs). The low level of political commitment to the cause of AMS owing primarily to the scarcity of AMS funding as well as the dearth of expertise in orchestrating AMS initiatives, are some of the biggest hurdles to AMS adoption among these countries (Kimang’a, 2012; Wertheim et al., 2013; Huttner et al., 2014; WHO, 2015). Such aspects remain under-addressed even with the recognition of these being the critical pre-requisites for effective AMS activities. In this context, LMICs are faced with the irony of few and/or defunct AMS programs despite being the hotspots for infectious diseases and antimicrobial resistance.

In Africa for instance, an international survey revealed the presence of AMS programs in only 14% of hospitals hence the lowest prevalence in comparison to all other regions (Huttner et al., 2014; Mendelson and Matsoso, 2015). This is further compounded by the fact that, most countries in the sub-saharan Africa have yet to implement any of the recommended interventions for resistance containment due to various economic and healthcare challenges (Okeke et al., 2007; Kimang’a, 2012). With this continent already confronting an overwhelming burden of communicable diseases (such as malaria, tuberculosis, and AIDS) while grappling with limited choice of quality antimicrobials for infection treatments, it is of little surprise that AMS has not been prioritized (Okeke et al., 2007). Surveillance has also been made difficult due to a combination of factors such as inadequate number of trained personnel and properly equipped laboratories (Kimang’a, 2012). This has hampered infection control/prevention endeavors that are already inadequate to begin with. The lack of clinical/epidemiological data has rendered the efforts to identify the pattern of resistance development and trend of spread futile hence complicates the devising of appropriately targeted interventions based on situational analysis.

## AMS at Odds with Healthcare Systems and Local Policies among the Emerging Economies

More pertinently, global AMS initiatives are simply incompatible with the less-refined healthcare systems and practices of many countries. Among the emerging economies, the accessibility to healthcare and pharmaceuticals has improved in tandem with economic progress with the caveat of high prevalence of inappropriate antibiotic use. This is evident from a recent study which revealed the high rates of antibiotic prescribing in the primary care settings of Malaysia even for self-limiting conditions (Rahman et al., 2016). Similarly, antibiotic dispensing without prescription/medical consultation is also a common practice as can be seen in countries like China, Chile, Malaysia, and Vietnam (Nguyen et al., 2013; Tiong et al., 2016). Self-medication is rampant, often being dubbed the most affordable and convenient mode of treatment (Nguyen et al., 2013; Huttner et al., 2014; Tiong et al., 2016). There is also demand for drugs such as antibiotics from the gray market, driven primarily by their cheaper prices, and in part by the poor regulatory enforcement. The risk of resistance due to irrational antibiotic use is further augmented by the consumption of counterfeit antibiotics with dubious pharmacokinetics profile since sup-optimal dosing is a prevalent cause for breeding human reservoirs of resistant pathogens (Bate et al., 2013). This has proved to be problematic in some parts of Africa, Asia, and Latin America where up to 30% of medicine on sales are counterfeit, exacerbated by the high frequency of incomplete antibiotic course among the lower income group whose antibiotic consumptions are for the purpose of symptomatic relief instead of microbial eradication (WHO, 2006; Okeke et al., 2007).

Moreover, the economic contributions of the pharmaceutical market as well as the need of such laxly monitored drug supply chain to serve as a form of affordable ‘system-of-care’ to the general population may post a regulatory dilemma to health authorities. This will, however, be a double-edged sword since such healthcare system has indirectly derailed the stewardship for the judicious use of antibiotics through its profit-driven model. The pharmaceutical industries in numerous developing- and developed-nations have been reported to incentivise health practitioners for pushing the sales of drugs including antibiotics (Tiong et al., 2016). This is worsened by the absence of a regulatory framework that separates prescribing and dispensing to elicit an effective system of checks and balances in the healthcare systems of this part of the world. Therefore, rational prescribing of antibiotics may be precluded by the monetary gains derived from the sales of drugs. Likewise, the under-addressed low level of public awareness pertaining to rational use of antibiotic is an important causative factor for patient -pressured antibiotic over-prescribing among doctors as well as the worrisome culture of antibiotic sharing (Stevenson et al., 1998; Hoffman et al., 2003; Ellis and Mullan, 2009). It will be an uphill task for AMS programs to achieve the intended objectives if national policies and prevailing local sentiments are not aligned with the concepts of AMS. Needless to say, any push to implement global AMS initiatives in these countries would first require strong political will to amend existing policies to make them AMS-friendly.

## High Income Nations are Not Responding Proactively to the Threat of Antimicrobial Resistance

The commitment to AMS among the developed nations could be further improved given their economic and technological prowess as well as the absence of various adversities faced by the low- and middle-income nations. These nations have long recognized antibiotic resistance as a global health threat although interventions have been gradual which have since been outpaced by the rate of resistance development and spread. An intensifying sense of urgency in recent years has seen numerous recommendations to boost AMS published by experts in the field, and it is heartening to see some have been taken forward although others remain unheeded hence the remaining gaps to be filled. Some governments are still perceived to be semi-committed to the cause of AMS due to their reluctance to prioritize the funding of national AMS and its related initiatives. Policymakers are perhaps mindful of the significant investment required to sustain AMS programs which are generally long term projects where positive outcomes are not guaranteed.

This appears to parallel the insufficient emphasis accorded to the important AMS-complementing research particularly those pertaining to bacteriology that may elicit crucial understanding of resistance mechanisms and patterns of spread. This is exemplified by several studies that revealed the paltry investment into the antimicrobial resistance research which constituted merely 3.9% of the total funding in the UK for infectious disease research between 1997–2010 whereas barely 1% of research funds across Europe were channeled into antibiotic research between 2008–2013 (Bragginton and Piddock, 2014; Head et al., 2014). The same is true in the United States where the US National Institute of Health allocated a meager 1.2% of its total research grant for antimicrobial resistance-related research between 2009–2014 (O’Neill, 2016). This is worrisome primarily when similar level of apathy was observed among the pharmaceutical companies toward the research and development of antibiotics. Not only is the capital investment into this area equally underwhelming (less than 5% of the total budget between 2003–2013), resources are continuously being diverted to areas which are deemed more commercially rewarding (O’Neill, 2016). If this trend continues, we may be facing an antibiotic apocalypse sooner than expected.

### Disorganized State of Current AMS

The lack of clear definition as to what constitutes an AMS initiative is likely to have led to an inaccurate estimate of the real prevalence of worldwide AMS activities (Huttner et al., 2014). This is attributable to the paucity of a global framework to guide the articulation of concerted action plans at both international and national levels, although this also raises the question as to the feasibility of some of the AMS activities established without the inputs of field experts.

There is a potential waste of funds due to the risk of non-validated activities eliciting limited impacts as can be seen in the implemented programs in some countries (Huttner et al., 2014). The greater concern, however, is the likelihood of deviation of hospital-specific AMS precepts from the fundamental rationales of national guidelines as the onus is placed on individual centers to formulate their specific measures based on their own understandings of AMS. This has led to the foreseeable unsynchronized and/or compartmentalized efforts which is complicated by the possible risk of concept divergence (Kaki et al., 2011). For instance, AMS may serve an excuse for some hospitals to limit antibiotic prescribing where the primary agenda is to cut cost (MacDougall and Polk, 2005). In light of this, it is always recommended for the analysis of the unintended consequence of AMS intervention to be conducted in conjunction to assessment of its effectiveness. Unfortunately, AMS feedback mechanisms remain inefficient, and are further hampered by possible reporting bias where only favorable outcomes are published in order to ensure continuous funding (MacDougall and Polk, 2005). All these are pointing to the disorganized state of current AMS endeavors due to the absence of standardized AMS policies. Nevertheless, it is worth noting that overly restrictive guidelines imposed with little justification may also result in poor voluntary compliance. Hence there is a need for inputs from all stakeholders (i.e., lawmakers, healthcare practitioners, scientists, economists, and other field experts) in order to establish a consensus-based practice guideline.

### Inter-Sectoral and International Fragmentation of AMS Implementation

It is irrefutable that the human health sector has become the subconscious focal point of AMS whereas the inappropriate use of antibiotics in areas like veterinary medicine and animal husbandry have often being overlooked. The absence of sectoral AMS coordination to guide simultaneous implementation may result in the breeding of resistant pathogens reservoirs in animals with risk of transmission to humans (Phillips et al., 2004; Martin et al., 2015). Even if some of the antibiotics are meant purely for veterinary use, the risk of development of cross-resistance with those used in humans cannot be discounted. Regrettably, *Salmonella enterica, E. coli* and *Enterococcus* spp resistant to sulphonamides and tetracycline in food animals and domestic pets have already been documented in South Africa (Mendelson and Matsoso, 2015). As such, the sectoral fragmentation in AMS remains a colossal issue to be addressed. Not all is well in human healthcare sector either. Positive outcomes of AMS are generally confined to countries with properly–planned and administered AMS programs such as those in the G7 block (Canada, France, Germany, Italy, Japan, UK, and USA; WHO, 2014, 2015; O’Neill, 2016). These countries are commendable for their proactive stance although it is immediately apparent that antibiotic resistance is a worldwide crisis which cannot be tackled by the efforts of few countries alone. While ‘success’ in the context of AMS-guided action plans remains difficult to define (hence beyond the scope of this article), it is safe to conclude that localized successes will have limited impact on a global scale. With the ease of global travel and trade, localized AMS achievements could be undone by the resistance transmitted from regions known to be ‘hotspots.’ In other words, common AMS policies between participating countries would be rendered ineffective in the absence of concurrent implementation across-the-board at the point of contact.

### The Way Forward

It is particularly welcoming to see the excellent recommendations made in the recently published *Review on Antimicrobial Resistance* for tackling antimicrobial resistance in a global manner (O’Neill, 2016). The authors of this article agreed with the published report that the fight against antimicrobial resistant-infections entails the forging of an international coalition involving all countries. A comprehensive framework for national plans has been established under WHO but we opine that it should be made legally binding in its push for simultaneous implementation across all sectors among member states whilst steering all toward a common precept. This is in-line with the integrative/collaborative approach advocated through the One Health concept which is receiving increasing attention lately (Mills, 2014). High income nations on the other hand, should assume a more proactive role and to take lead in administering the action plans internationally through the sharing of expertise, experience, and resources. In this context, it is paramount to expand the coverage of effective AMS initiatives to all LMICs in which implementation has proved problematic thus far due to the presence of many country-specific limiting factors. Attention should be paid in intensifying efforts in these nations whose populace made up approximately two third of world population, where a drug-resistant outbreak would most certainly set off far-reaching health and economic implications. The Fleming Fund is certainly a move in the right direction in providing impetus to improve drug-resistance infections surveillance in these countries (O’Neill, 2016). A global AMS initiatives fund with mandatory reporting of process indicators should also be considered to spur AMS programs particularly among the LMICs. Nonetheless, the immediate agenda for the international communities would be the identification of sources of sustainable funding in order to ensure the continuum of AMS initiatives worldwide.

It is encouraging to see the recent efforts in strengthening AMS among some of the emerging economies. The South African Antibiotic Stewardship Programme (SAASP) and Vietnam Resistance (VINARES) are two initiatives to be emulated by developing nations faced with the many adversities in combating antimicrobial resistance (Nguyen et al., 2013; Mendelson and Matsoso, 2015). Both SAASP and VINARES projects represent exemplary models of policy-guided national action plans formulated based on situational analysis, taking into account the resource-limiting environment of developing nations. It is important to note that, a dynamic approach should be taken where tweaking of AMS programs may be required from time to time in order to tailor the right initiatives to the local sentiments.

## Conclusion

We have certainly come a long way with our efforts in combating the scourge of antimicrobial resistance through AMS. Nevertheless, uncertainty remains as to how widespread is the global adoption of AMS in order to avert an impending world disaster. From a global perspective, AMS has yet to attain its intended objective in tackling worldwide spread of antimicrobial resistance. Hence, we should acknowledge that there are still gaps to be bridged especially in ensuring that future initiatives will be made more feasible, robust, and multi-faceted for wider adoption across different geographical and socio-economic settings. That being said, much time has been spent strategizing and it is time we translate these strategies into actions before the window of opportunity is lost.

## Author Contributions

JT, JL, and C-WM contributed toward drafting, revising, and completing of the manuscript.

## Conflict of Interest Statement

The authors declare that the research was conducted in the absence of any commercial or financial relationships that could be construed as a potential conflict of interest.
